# Anisotropic Dirac Fermions in BaMnBi_2_ and BaZnBi_2_

**DOI:** 10.1038/s41598-018-33512-w

**Published:** 2018-10-17

**Authors:** Hyejin Ryu, Se Young Park, Lijun Li, Weijun Ren, Jeffrey B. Neaton, Cedomir Petrovic, Choongyu Hwang, Sung-Kwan Mo

**Affiliations:** 1 Advanced Light Source, Lawrence Berkeley National Laboratory, Berkeley, CA 94720 USA; 2 Max Planck POSTECH Center for Complex Phase Materials, Pohang University of Science and Technology, Pohang, 37673 Korea; 3 Center for Spintronics, Korea Institute of Science and Technology, Seoul, 02792 Korea; 4 Department of Physics, University of California, Berkeley, CA 94720 United States; 5 Condensed Matter Physics and Materials Science Department, Brookhaven National Laboratory, Upton, New York 11973 United States; 6 Shenyang National Laboratory for Materials Science, Institute of Metal Research, Chinese Academy of Sciences, Shenyang, 110016 China; 7 Molecular Foundry, Lawrence Berkeley National Laboratory, Berkeley, California 94720 United States; 8 Kavli Energy Nanosciences Institute at Berkeley, Berkeley, California 94720 United States; 9 Department of Physics, Pusan National University, Busan, 46241 Korea

## Abstract

We investigate the electronic structure of BaMnBi_2_ and BaZnBi_2_ using angle-resolved photoemission spectroscopy and first-principles calculations. Although they share similar structural properties, we show that their electronic structure exhibit dramatic differences. A strong anisotropic Dirac dispersion is revealed in BaMnBi_2_ with a decreased asymmetry factor compared with other members of AMnBi_2_ (A = alkali earth or rare earth elements) family. In addition to the Dirac cones, multiple bands crossing the Fermi energy give rise to a complex Fermi surface topology for BaZnBi_2_. We further show that the strength of hybridization between Bi-*p* and Mn-*d*/Zn-*s* states is the main driver of the differences in electronic structure for these two related compounds.

## Introduction

Dirac materials, characterized by the linear dispersion of their low-energy quasi-particle excitations, have received significant recent attention, given their potential to host various exotic phenomena such as high mobilities due to strongly suppressed backscattering, unconventional quantum Hall effects, and Klein tunneling^1,2^. Moreover, the linear crossing around the Fermi level (*E*_F_) is observed in broad categories of materials of strong contemporary interest, including topological insulators, *d*-wave superconductors, and iron-based compounds^3^.

Anisotropic Dirac materials are distinguished by their strong momentum-dependent Fermi velocities on the Dirac cone^4,5^. The anisotropy enables the electrons to propagate differently depending on crystallographic direction, providing additional versatility for applications^6^. Significant effort in prior work has been undertaken to generate the anisotropy on Dirac cone using patterned superstructures, mechanical stress, and heterostructures, among others^7–12^.

Recently, a new family of materials, AMnBi_2_ (A = alkaline earth or rare earth elements), was reported to have intrinsic anisotropy in their Dirac dispersion. The role of the A-site cation on the Dirac dispersion has been studied extensively, where the dispersion has been shown to qualitatively change upon breaking mirror symmetry between Ca- and Sr-based AMnBi_2_ compounds^13^ or breaking time reversal symmetry inducing the Weyl semimetallic phase in Yb-based AMnBi_2_ compounds^14^. However, direct measurements of the electronic structure of the Ba-based AMnBi_2_ compound have yet to be reported; and BaMnBi_2_ would aid in furthering a systematic investigation of the relation between A-site ionic radii and the nature of the Dirac dispersion. More importantly, it has been shown by first-principles calculations that the partially filled Mn-*d* states do not directly contribute states near the *E*_F_^13^, unlike many other transition metal pnictides; yet the Mn atoms are known to possess net magnetic moments in the *d*^5^ high-spin state, possibly giving rise to an anomalous magnetoresistance^15^. Investigating the role of the transition metal *d*-states on low energy Dirac dispersion is challenging due to the multi-valence nature of these ions; the change in the orbital occupancy may induce a variety of ordered states and accompanying atomic distortions. To avoid such structural distortions from the *d*^5^ (Jahn-Teller inactive) configuration that lower symmetry, one must consider B-site ions with empty or fully filled *d* bands, such as AZnBi_2,_ as we do below.

In this report, we explore the electronic structure of BaMnBi_2_ and BaZnBi_2_ using angle-resolved photoemission spectroscopy (ARPES) and first-principles density functional theory (DFT) calculations. We find that using Ba on the A-site leads to anisotropic Dirac bands, although with different asymmetry and anisotropy from (Ca,Sr)MnBi_2_ compounds. In addition, substantial changes in the electronic structure are found by substituting Zn with Mn (3*d*^5^ with Mn^2+^ to 3*d*^10^ with Zn^2+^, respectively), yielding more trivial bands at *E*_F_ due to the increased hybridization between Zn and Bi states.

**Figure 1:**
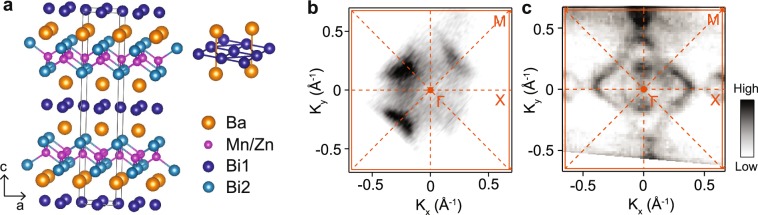
Crystal structures and Fermi surfaces. (**a**) Crystal structures of BaMnBi_2_ and BaZnBi_2_. Ba and Bi-net structure illustrated on the upper right corner is similar to SrMnBi_2_ in the same space group, I4/mmm. The black solid line indicates the conventional unit cell. (**b**,**c**) Fermi surfaces of BaMnBi_2_ (**b**) and BaZnBi_2_ (**c**) measured by ARPES. Brillouin zone and high symmetry lines are marked by orange solid and dotted lines, respectively. Γ, X, and M are the high symmetry points.

Figure 1 shows the atomic structure of BaMnBi_2_ and BaZnBi_2_. These materials consist of alternating layers of Bi-net and Mn (or Zn)-Bi tetrahedra separated by Ba layers located in mirror symmetric positions with respect to the Bi-net (upper right panel of Fig. 1a), satisfying *I4*/*mmm* space group symmetry. Table 1 compares the lattice constants and ionic radii of A-site ions for other AMnBi_2_ compounds with BaZnBi_2_. With A-site cations of smaller ionic radius, the atomic structure takes up the *P4*/*nmm* space group, in which the A-site cations are located at staggered positions, breaking the mirror symmetry with respect to the Bi square net^13^. We find by X-ray diffraction that the space group of both BaMnBi_2_ and BaZnBi_2_ are *I4*/*mmm*, confirming the sensitivity of the space group to A-site. Moreover, for AMnBi_2_ compounds with the *I4*/*mmm* space group, the *c*/*a* ratio is measured to increases with increasing A-site ionic radius, as expected. A unique feature of the crystal structure associated with this family of materials is that there are two types of Bi atoms in the unit cell: one consisting of the Bi-net (defined as Bi1) and the other forming the Bi-tetrahedra around the Mn (or Zn) cations (defined as Bi2). It has been pointed out that states near *E*_F_ of AMnBi_2_ compounds are dominated by Bi1-*p* orbitals whereas the Bi2-*p* states that hybridize with the Mn-*d* orbitals are away from the Fermi level^13^. We note that the *c*/*a* ratio of the BaZnBi_2_ is dramatically smaller than those reported for the Mn family, a reduction that can be primarily attributed to the reduced Zn-Bi distance and that reflects differences in the nature of bonding between Zn-Bi and Mn-Bi. With the small binding energy of the Bi2-*p* states in AMnBi_2_^13^, the expected change in the bonding character in BaZnBi_2_ should induce a significant change in the states near the *E*_F_, which will be discussed later in our ARPES data and first-principles results.

**Table 1:** Structural properties of AMnBi_2_ (A = Ca,Yb,Eu,Sr,Ba) and BaMnBi_2_.

Space Group	CaMnBi_2_^25^	YbMnBi_2_^14^	EuMnBi_2_^26^	SrMnBi_2_^27^	BaMnBi_2_^27^	BaZnBi_2_
	*P4*/*nmm* (129)		*I4*/*mmm* (139)			
*a*-lattice constant (Å)	4.50	4.48	4.53	4.58	4.63	4.86
*c*-lattice constant (Å)	11.08	10.80	22.43	23.13	24.22	21.99
*c*/*a* ratio	2.46	2.41	4.95	5.05	5.23	4.52
Mn(Zn)-Bi distance (Å)	2.88	2.87	2.87	2.89	3.39	2.81
A-ionic radius (pm)	114	116	131	132	149	149

Fermi surfaces (FSs) of BaMnBi_2_ (Fig. 1b) and BaZnBi_2_ (Fig. 1c) measured by ARPES show notably distinct features. The FS of BaMnBi_2_ displays four crescent-shape hole pockets resulting from anisotropic Dirac bands along Γ-M directions, typical of the other anisotropic Dirac materials AMnBi_2_ (A = Ca, Sr, Eu, and Yb) of this class^14,16^. On the other hand, the BaZnBi_2_ FS shows new features, including double layers of large diamond-like pieces connecting the four X points in the Brillouin zone (BZ) and two concentric circle-like hole pockets around the Γ point. The four corners of the diamond-like FS features overlap with neighboring ones at the X points, generating additional small diamond-like electron pockets at these four X points in the BZ.

**Figure 2:**
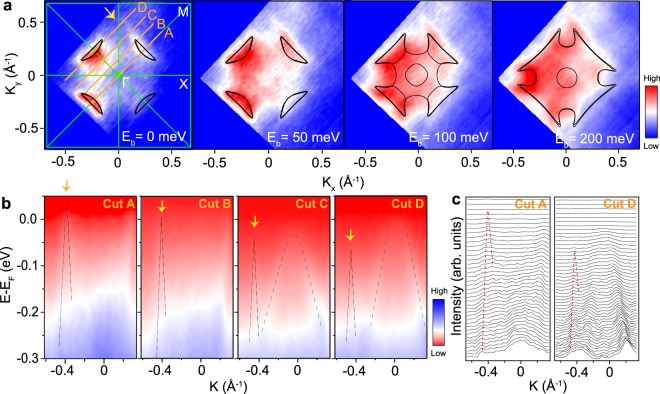
Electronic structure of BaMnBi_2_. (**a**) Constant energy contours for different binding energy with DFT calculations drawn by black lines (Fermi level shifted by −0.05 eV for better comparison) overplayed on top of ARPES constant energy map. Brillouin zone and high symmetry lines are marked by green lines. Γ, X, and M are the high symmetry points. (**b**) Electronic structures along various momentum directions (A–D) labeled using orange lines on (**a**). The yellow arrows on (**a** and **b**) indicate the locations of the Dirac bands in momentum space on the FS and four cuts parallel to the Γ-M direction. These Dirac bands are represented by two black straight dotted lines as a guide to eyes. We determined the Dirac band crossing point as the intersection of those two lines. (**c**) Momentum distribution curves for cut A and cut D with red dotted lines as a guide to eyes.

**Figure 3:**
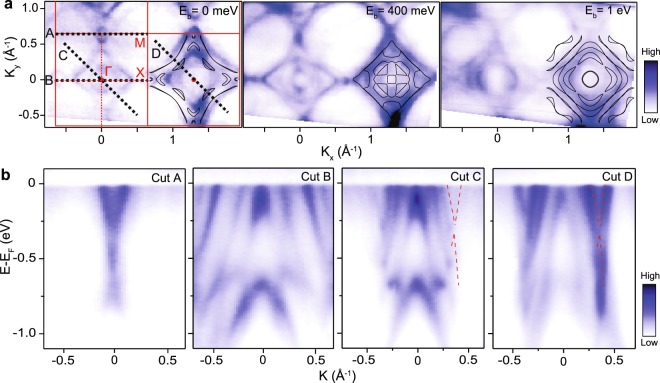
Electronic structure of BaZnBi_2_. (**a**) Constant energy contours for different binding energy with DFT calculation results (black solid lines) in which the Fermi energy is shifted by 0.4 eV and eigenvalues are renormalized by 1.3 for better comparison. Brillouin zone and high symmetry lines are marked by red lines. Γ, X, and M are the high symmetry points. (**b**) Electronic band dispersions along several different momentum directions (A–D) drawn by black dotted lines on (**a**). Red dotted lines are a guide to eyes for Dirac bands.

Further investigations of the electronic structures of BaMnBi_2_ and BaZnBi_2_ from our ARPES measurements are illustrated on Figs 2 and 3, respectively. Constant energy contours of BaMnBi_2_ at different binding energies are shown in Fig. 2a. As the binding energy increases, the size of four crescent-shaped iso-energy surface also increases, indicating the hole-like nature of anisotropic Dirac bands in Γ-M directions. Meanwhile, the other four sets of new crescent-like pockets around the X point start to appear around binding energy E_B_ = 100 meV. Our first-principles DFT calculations within the generalized gradient approximation (GGA) and including spin-orbit (SO) interactions, shown with black solid lines on Fig. 2a, predict dispersion consistent with the experimental data.

To analyze these anisotropic Dirac bands further, we examine the measured electronic band dispersion along four cuts from A to D (Fig. 2b). The Dirac bands marked by yellow arrows exhibit a clear asymmetry with a steeper left branch of the band compared to the right one (Fig. 2b,c). The Fermi velocity of the left (*v*_L_) and the right (*v*_R_) branches are ~6 eV·Å and ~1.9 eV·Å, respectively, which yields an asymmetry value ((*v*_L_ − *v*_R_)/*v*_L_) ~0.68. This is smaller than the asymmetry values of CaMnBi_2_ (0.80) and SrMnBi_2_ (0.78)^16^. It is well known that this asymmetry originates from the strong spin-orbit coupling which also gaps out the Dirac band^13,16^. The doubly-degenerate Dirac cones associated with the low energy electronic structure along Γ-M and absence of states along Γ-X direction is consistent with our first-principles calculations, which will be discussed in more detail below.

The crossing points of Dirac bands are the highest in energy along Γ-M directions and shift downwards with momentum away from the Γ-M directions (from cut A to cut D). At the same time, on cut C and D, the Dirac band dissected along the perpendicular direction compared to the cut A appears around the Γ point. The slope of the Γ-point Dirac band is much less steep compared to the one in cut A, which clearly exhibits the strong anisotropic characteristics expected from a Dirac band. For a quantitative analysis of the anisotropy of Dirac bands, we extract the Fermi velocity along Γ-M (*v*_∥_ = (*v*_L+_ *v*_R_)/2) and perpendicular to Γ-M (*v*_⊥_), which yield ~4 eV·Å and ~0.3 eV·Å, respectively. The anisotropy value can be defined as *v*_∥_/*v*_⊥_ ~ 13, which is smaller than CaMnBi_2_ (64), YbMnBi_2_ (209), but roughly equivalent to SrMnBi_2_ (13)^14,16^. This illustrates a correlation of the strength of the anisotropy with space group, since CaMnBi_2_ and YbMnBi_2_ with *P4*/*nmm* display relatively stronger anisotropy than SrMnBi_2_ and BaMnBi_2_ that belong to *I4*/*mmm*.

We substitute Mn with Zn to investigate the effect of their different valence configurations on the band structure and anisotropy. The constant energy contours measured for BaZnBi_2_ (Fig. 3a) display significant differences in the band features compared to those of BaMnBi_2_ (Fig. 2a) with two concentric hole-like inner circular FS pockets surrounded by two concentric hole-like outer diamond pockets with electron-like FS at the X point that originate from band overlap. As the binding energy increases, circular and diamond bands expand and overlap with each other while the pockets at the X point shrink, as expected, from the binding energy-dependent nature of the hole-like and electron-like bands, respectively. The FS calculated from our first-principles DFT-GGA + SO calculations reproduce the main features, such as the increase in the number of bands crossing the *E*_F_ and diamond-shaped bands; our calculations also show deviations compared with ARPES spectra, potentially due to missing correlation effects beyond DFT, indicated by a substantial band renormalization about 1.3 used for better agreement between the DFT and ARPES band structures (see the discussion in the calculation methods).

**Figure 4:**
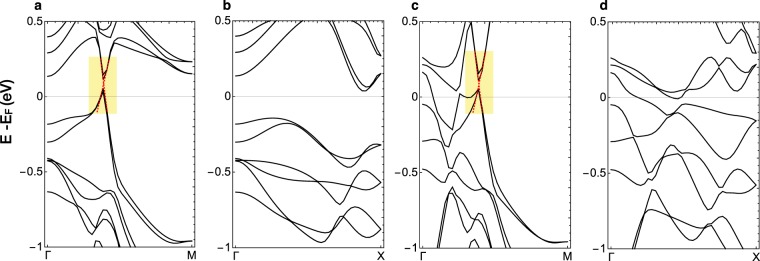
First-principles band structures of BaMnBi_2_ and BaZnBi_2_. (**a**,**b)** Electronic band dispersion of BaMnBi_2_ along high symmetry lines. (**c**,**d**) Electronic band dispersion of BaZnBi_2_ along high symmetry lines. Yellow boxes denote the low energy anisotropic linear dispersions and the red dashed lines represent least square fits to the linear dispersion.

**Figure 5:**
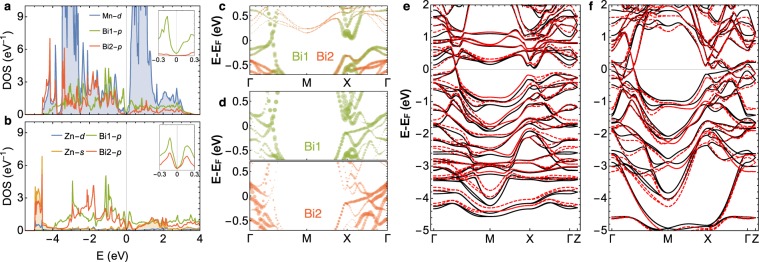
Projected density of states (PDOS), orbital-projected band structures, and electronic band structures of BaMnBi_2_ and BaZnBi_2_ by first-principle calculations. (**a**) PDOS for Mn-*d* and Bi-*p* orbitals of BaMnBi_2_. (**b**) PDOS for Zn-*d*, Bi-*p*, and Zn-*s* orbitals of BaZnBi_2_. The inset figures in the panel a and b are the magnification of PDOS of Bi1-*p* and Bi2-*p* states around the Fermi level. (**c**,**d**) Band structures around the Fermi level of BaMnBi_2_ (panel c) and BaZnBi_2_ (panel d) projected for Bi1-*p* (red circles) and Bi2-*p* (green circles) orbitals. Orbital contribution is proportional to the radius of each point. (**e**) Band structures of BaMnBi_2_ calculated with the relaxed atomic structure of BaMnBi_2_ (black solid lines) and with that of BaZnBi_2_ (red dashed lines). (**f**) Band structures of BaZnBi_2_ calculated with the relaxed atomic structure of BaZnBi_2_ (black solid lines) and with that of BaMnBi_2_ (red dashed lines).

The first-principles band dispersions of BaMnBi_2_ and BaZnBi_2_ along high symmetry lines are shown in Fig. 4. Panels a and b show band dispersions of BaMnBi_2_ and exhibit degenerate Dirac cones along the Γ-M direction and absence of the states along the Γ-X direction, consistent with the experimental data around the Fermi level. The steep dispersion at the Dirac cone originates with the Bi-*p* orbitals arranged in the square net, as shown from our calculated projected band dispersions (Fig. 5c), combined with half-filled *p*_*x*_ and *p*_*y*_ bands^13^. We calculate Fermi velocities along the Γ-M direction by fitting to the linear dispersion (Fig. 4a). The left and right Fermi velocity are 8.1 eV·Å and 4.4 eV·Å, respectively, with an asymmetry value of 0.46, showing significant band renormalization of 1.3 for *v*_L_ and 2.3 for *v*_R_. The band structure of BaZnBi_2_ presented in Fig. 4d,e clearly shows the increase in the number of bands crossing the *E*_F_, indicates a large direct gap around the M-point, and the decrease in the direct gap near the X-point, reproducing important features of the experimental data. The calculated *v*_L_ and *v*_R_ are 8.1 eV·Å and 4.4 eV·Å with an asymmetry value 0.4, insensitive to the substitution of the transition metal ion.

In order to investigate the origin of the changes in the electronic structure associated with substituting Mn for Zn, we calculate and compare the projected density of the states (PDOS) of BaMnBi_2_ and BaZnBi_2_ (see Fig. 5a,b). In BaMnBi_2_, the Bi2-*p* states hybridize primarily with Mn-*d* shown from the overlap in the computed PDOS around −4.5 eV; whereas Bi2-*p* states in the Zn compound hybridize mainly with the Zn-*s* states over a broad energy range, from around −5 eV to 0–2 eV above *E*_F_. Since the bonding between the Zn-*s* and Bi2-*p* orbitals is stronger than Mn-*d* and Bi2-*p* orbitals due to the large spatial extent of the Zn-*s* orbitals and shorter Zn-Bi distance (see Table 1), the antibonding Bi2-*p* bands shifts upwards in BaZnBi_2_ compared with BaMnBi_2_. The upward shifts in the Bi2-*p* bands result in additional band crossings at *E*_F_, shown by orbital-projected band structures in Fig. 5c,d. The Bi2-*p* states around the Γ point in BaZnBi_2_ move up by about 0.5 eV compared with those in the Mn compound, resulting in Bi2-*p* derived bands crossing *E*_F_.

In order to distinguish between the effect of the change in structure (Mn/Zn to Bi distance) and the change in bonding character (*d-p* vs. *s-p* hybridization), we compare the band structures of BaMn(Zn)Bi_2_ calculated with relaxed atomic structure of BaMn(Zn)Bi_2_ and with deliberate changes of Mn(Zn) – Bi distance for that of BaZn(Mn)Bi_2_ in Fig. 5e,f. The results demonstrate that there is little difference in the band dispersion due to the change in structure. Thus, we can conclude that the change in the band structure is mainly caused by the difference in bonding character. Our calculations also suggest that, to isolate the Dirac bands crossing *E*_F_, it would be ideal to choose transition metal ions with frontier orbitals that hybridize weakly with the Bi2-*p* orbitals and that are gapped at the Fermi level.

In conclusion, we have investigated the electronic structure of BaMnBi_2_ and BaZnBi_2_ using ARPES and first-principles calculations, focusing on the effect of substituting A-site cation with larger ionic radius and the role of the transition metal states on the band dispersion near *E*_F_. Compared with the isostructural compound SrMnBi_2_, substitution of the A-site cation with larger ionic radius results in a small decrease in the spin-orbit induced asymmetry and negligible change in the anisotropy of the Dirac cone, and maintains the same Fermi surface topology originating with the Bi-*p* states from the square net. However, we find that the substitution of the Mn with Zn gives rise to a drastic change in the dispersion near the Fermi level with Bi-*p* states derived from Bi square net and Zn-Bi complex due to the large hybridization between Zn-*s* and Bi-*p* states. Our results imply that transition metals with frontier orbitals weakly hybridizing with Bi-*p* states may be ideal for the isolation of the Dirac bands crossing at the Fermi level.

## Method

### Single crystal growth

Single crystals of BaMnBi_2_ and BaZnBi_2_ were grown from molten metallic fluxes as described previously^15,17^.

### ARPES measurement

ARPES measurements were performed at the HERS endstation of the Beamline 10.0.1, Advanced Light Source, Lawrence Berkeley National Laboratory. The ARPES system was equipped with a Scienta R4000 electron analyzer under the base pressure 3 × 10^−11^ Torr. The photon energy was set at 60 eV for BaMnBi_2_ and 57.5 eV for BaZnBi_2_ with energy and angular resolution of 25 meV and 0.1 degree. The choice of photon energy was based on the most counts and the best contrast for the spectra, since there exist negligible *k*_*z*_ dispersions in our ARPES measurements and first principle calculations. Measurements were made at the temperature 15 K.

### Electronic structure calculations

We perform first-principles density functional theory calculations with the generalized gradient approximation (GGA) method using the Vienna *ab-initio* simulation package^18,19^. The Perdew-Becke-Erzenhof (PBE) parametrization^20^ are used for the GGA exchange correlation functional. Spin-orbit coupling is included self-consistently for all the calculations. We use the projector augmented wave method^21^ with an energy cutoff of 500 eV and *k*-point sampling on a 6 × 6 × 2 grid. The atomic positions are fully relaxed until Hellmann-Feynman forces are less than 0.02 eV/Å. The lattice constants relaxed with the PBE functional are in good agreement with experiment for BaMnBi_2_ with difference of 1.5% for volume and −0.04% for *c*/*a* ratio. For BaZnBi_2_ the unit-cell volume calculated by the GGA shows reasonable agreement with 2.3% error with respect to the experimental value but the *c*/*a* ratio deviates more significantly, with an error about 8.5% compared with experimental value. The deviation in the *c*/*a* ratio does not change significantly with the inclusion of the long-range Coulomb interaction using hybrid functional^22^ but decreases with van der Waals interaction (vdW-D3)^23^ to 5%. Since there are only small changes in the band structures by including vdW, the band structures using GGA exchange correlation functional are presented both BaMnBi_2_ and BaZnBi_2_. The band structures of BaMnBi_2_ are calculated with checkerboard type of antiferromagnetic ordering for the Mn-*d* spins; the checkerboard order is calculated to have the lowest total energy which is 0.08 and 0.26 eV per formula unit lower than stripe-type antiferromagnetic ordering and ferromagnetic ordering, respectively, consistent with other AMnBi_2_ compounds^13,24^. Fermi surfaces are calculated by interpolating energy dispersion using dense *k*-grid points (80 × 80) at *k*_*z*_ = 0.
